# Classification for avian malaria parasite *Plasmodium gallinaceum* blood stages by using deep convolutional neural networks

**DOI:** 10.1038/s41598-021-96475-5

**Published:** 2021-08-19

**Authors:** Veerayuth Kittichai, Morakot Kaewthamasorn, Suchansa Thanee, Rangsan Jomtarak, Kamonpob Klanboot, Kaung Myat Naing, Teerawat Tongloy, Santhad Chuwongin, Siridech Boonsang

**Affiliations:** 1 Faculty of Medicine, King Mongkut’s Institute of Technology Ladkrabang, Bangkok, Thailand; 2 Veterinary Parasitology Research Unit, Faculty of Veterinary Science, Chulalongkorn University, Bangkok, Thailand; 3 Faculty of Science and Technology, Suan Dusit University, Bangkok, Thailand; 4 College of Advanced Manufacturing Innovation, King Mongkut’s Institute of Technology Ladkrabang, Bangkok, Thailand; 5 Department of Electrical Engineering, School of Engineering, King Mongkut’s Institute of Technology Ladkrabang, Bangkok, Thailand

**Keywords:** Mathematics and computing, Pathology, Parasitology

## Abstract

The infection of an avian malaria parasite (*Plasmodium gallinaceum*) in domestic chickens presents a major threat to the poultry industry because it causes economic loss in both the quality and quantity of meat and egg production. Computer-aided diagnosis has been developed to automatically identify avian malaria infections and classify the blood infection stage development. In this study, four types of deep convolutional neural networks, namely Darknet, Darknet19, Darknet19-448 and Densenet201 are used to classify *P. gallinaceum* blood stages. We randomly collected a dataset of 12,761 single-cell images consisting of three parasite stages from ten-infected blood films stained by Giemsa. All images were confirmed by three well-trained examiners. The study mainly compared several image classification models and used both qualitative and quantitative data for the evaluation of the proposed models. In the model-wise comparison, the four neural network models gave us high values with a mean average accuracy of at least 97%. The Darknet can reproduce a superior performance in the classification of the *P. gallinaceum* development stages across any other model architectures. Furthermore, the Darknet has the best performance in multiple class-wise classification, with average values of greater than 99% in accuracy, specificity, and sensitivity. It also has a low misclassification rate (< 1%) than the other three models. Therefore, the model is more suitable in the classification of *P. gallinaceum* blood stages. The findings could help us create a fast-screening method to help non-experts in field studies where there is a lack of specialized instruments for avian malaria diagnostics.

## Introduction

Avian malaria, a mosquito-borne disease, is one of the most common veterinary threats in tropical regions, including South East Asia and South Asia^1^. *Plasmodium gallinaceum* is an important causative agent of avian malaria, which causes more than 80% mortality if left untreated^2^. The disease entails an economic and agricultural loss in poultry processing systems such as poor quality and quantity of meat and egg production^1^. Early and rapid routine screening for a safe and low-cost parasite infection may help prevent transmissions of the disease. Microscopic inspection is a gold standard approach and is widely used to examine and identify avian malaria-infected blood stages under thin-blood film examinations, the outcome of which is validated by highly trained and qualified technicians^3^. Nevertheless, the precise outcome of the above-mentioned procedure depends on the consistency of the blood smearing and staining process. In addition, asymptomatic with low parasite infection can be time-consuming and may be undetectable or questionable as a result of inter/intra examiner variability^3^. In addition, unspecific clinical symptoms of avian malaria, such as anorexia, anemia, and green stools, are often seen^1,4^. While most of the nucleic acid-based amplification processes, such as the polymerase chain reaction (PCR) assay, is an efficient method with high sensitivity and malaria detection specificity, it requires an optimal consistency of genomic DNA and expensive tools such as thermocycler and electrophoresis apparatus^5–7^. A professional molecular biologist is also required for further verification of the analysis. Consistently, the molecular biology approach could not always be affordable in low-income and resource-limited nations. As a result, an automated identification tool is desired.

Artificial intelligence (AI) technology is currently a positive integration success in a diverse areas of interest, including agriculture, medicine and veterinary medicine^8–15^. Computer-aided diagnosis, an AI subdivision, has been developed to identify human malaria infections and to classify their blood stage of the parasite growth. These can be used to assist clinical decision-making. Machine learning applications have also been studied in the documented veterinary field^16^. Previous research has suggested a diagnostic tool in veterinary medicine focused on image analysis using machine learning techniques^17,18^, such as microscopic examination used to help diagnose disorder and disease in fish farm^19^. The analysis referred to above is intended to enhance the inspection of the pathogen region in the image by means of object detection, which includes various image processing techniques, including noise reduction, edge detection, morphological operations and context extraction.

Deep learning is a revolutionary and groundbreaking approach that has been incorporated into microscopic analysis for the veterinary medicine field^20,21^. The methods are combined and tailored for individual datasets that differ in the size of the region of interest. In specific, deep learning technology is applied to end-to-end methods of extraction of features and self-discovery. The deep learning algorithm is very popular and useful with the emergence of a high-power computing machine to be used to study the classification of images and the recognition of clinical issues. Several neural network models have been used to contend with the animal sector, the Single-Shot MultiBox Detector (SSD) model used to evaluate the percentage of reticulocytes in cat’s samples^22^, Alexnet for classification of fish disease such as Epizootic ulcerative syndrome (EUS), Ichthyophthirius (Ich) and Columnaris^19^. Deep learning is a revolutionary and groundbreaking approach that has been incorporated recently into microscopic analysis for the veterinary medicine field. The work described above shows that deep learning algorithms can be applied successfully in the field of veterinary medicine.

Previously, several techniques for image-based identification and classification of malaria parasite infections have been discussed, such as dark stretching technique^23^, modified fuzzy divergence technique, segmentation techniques^24^, adaptive color segmentation and tree-based decision classification^25^, segmentation, feature extraction, and SVM classifier^26^, convolutional neural classification^27,28^. Moreover, deep CNN research has been conducted under the channel color space segmentation method^29^, deep belief network technique^30^, the Faster Region-based Convolutional Neural Network (Faster R-CNN)^31^, and the MATLAB-based Zach Threshold process for segmentation technique^32^. Successfully, several studies have reported the use of deep learning models to classify malaria parasites as automatic, rapid, and accurate approaches^33–35^. Interestingly, more than 95% of the accuracy is recorded in the detection of malaria-infected cells using three well-known CNNs, including LeNet, AlexNet, and GoogLeNet^36^. The previous work demonstrated micro-platforms to study and identify the infected avian red blood cells by using morphological modifications on the RBC surface to reveal the phases of *P. gallinaceum*. Since malaria has been described as a disease of blood and blood-forming tissues, the blood film sample has been diagnosed to better understand different degrees of disease^37^. Early rapid screening of parasite infection with reliable and also low-cost development is required, which could help us deter the spread of the disease. Therefore, timely identification of the malaria parasite in a blood smear test is crucial because it needs reliable and early diagnosis for successful containment.

A hybrid platform (VGG19 and SVM) recently demonstrated high performance in detecting infected and non-infected malaria parasite images, as observed follows: 93.44 per cent sensitivity; 92.92 per cent specificity; 89.95 per cent precision, 91.66 per cent F-score and 93.13 per cent accuracy^38^. The outstanding performance of the hybrid algorithms mentioned previously motivates us to develop a hybrid object detection and classification method for further classifying avian malaria in Thailand.

In this work, we employ two-state learning techniques, which combine an object detection model based on YOLOv3 with one of four classification models, namely Darknet, Darknet19, Darknet19-448, and Densenet201, to characterize the avian malaria blood stages of *P. gallinaceum*. The primary contribution is a comparison of several image classification methods utilizing blood protozoa images as qualitative and quantitative data for evaluating the proposed models. We compared the effectiveness of the proposed model in predicting parasitized and healthy chicken RBCs in thin blood film images. Furthermore, the suggested model can predict the stage of malaria development, which impacts both the severity of illness and the likelihood of malaria transmission. Because of the medicinal relevance of human malaria parasites, this type of technique has been used more extensively. To the best of our knowledge, this is the first study to use CNNs deep learning model in the categorization of clinical datasets related to clinical issues in *P. gallinaceum*-infected chickens. This work contributes significantly to the field since conventional identification relies heavily on microscopist experts. Such experts require years of training and practice. Therefore, it would be very helpful to have CNNs deep learning aid and could be done by technicians without intensive training.

## Methods

### Ethics statement

Archived Giemsa-stained thin-blood films have been collected from previous studies^1,39^. This study was reviewed and approved by the Chulalongkorn University Faculty of Veterinary Science Biosafety Committee (Approval No. 1931011) and was approved by the Institutional Animal Care and Use Committee in accordance with university regulations and policies governing the care and use of laboratory animals (Approval No. 1931091). In this study, we strictly adhered to the university's relevant guidelines and regulations. All procedures were carried in accordance with the ARRIVE guidelines.

### Data collections

In the present study, blood films were prepared immediately after withdrawal of blood samples from the infected chickens. This results in the proper shape of an erythrocyte’s nuclei. The parasite infection was confirmed by three well-trained investigators using microscopic diagnosis. In addition, all *P. gallinaceum* positive slides used in this study were also confirmed by PCR and sequencing as described in Xuan et al.^40^. Ten *P. gallinaceum*-infected chicken blood films were randomly chosen. A total of 432 images of chicken blood cells at various stages of malarial growth were taken at 1000 × using an oil immersion magnification mounted to a light microscope (Olympus CX31, Tokyo, Japan).The digitized chicken blood cells were captured using an Olympus DP21-SAL digital camera (Tokyo, Japan). An individual RBC image from each blood film was selected to be used in an image with a region of interest to the so-called monolayer area.

### A hybrid two-stage model: RBC detection and classification models

The proposed methodology for classifying the blood stages of avian malaria, *P. gallinaceum*, uses a hybrid two-stage model (object identification YOLOv3 and Darknet/Densenet201 classification algorithms). Previously, the combination of two CNN models was reported to have increased prediction accuracy^41,42^. It was more beneficial when a combination of two deep learning models such as using two-state learning strategies of concatenated YOLO models for identifying genus, species and gender of the mosquito vector^42^. In this work, the first stage of the proposed model is the YOLOv3-based object detection, which aims to distinguish a single RBC image from those inside a microscopic image. Among other object detection models, the YOLOv3 model outperformed the others, in terms of localization and classification accuracy^14^. Model inference, in particular, can be accomplished in real-time by processing 45–155 frames per second. It can also recognize up to 9000 object classes. After encoding the circumstantial information of the relative classes, the model can detect the entire area of an input image set with only a few false positives^43^ and shows a balance of accuracy and speed in practical applications that time is not restrictive^44^.

Cropped images (single RBC) from its first stage model inference were used as inputs for the second stage model. The classification model was used in the second stage to categorize the single RBC detected. CNN model candidates were widely used for studying image classification, from a top-5 pre-trained model which was measured as single-crop validation accuracy at 94.7%^45^. The models employed in this study are the top ranking of the ILSVRC2016 ImageNet competition to identify pathological tasks. These versions are in the following sizes; Densenet201, Darknet, Darknet19, and Darknet19-448^45^. The model prediction was automatically masked and colored with a JET color map generated by the Class Activation Map (CAM) algorithm^46^.

### Dataset preparation

**Figure 1:**
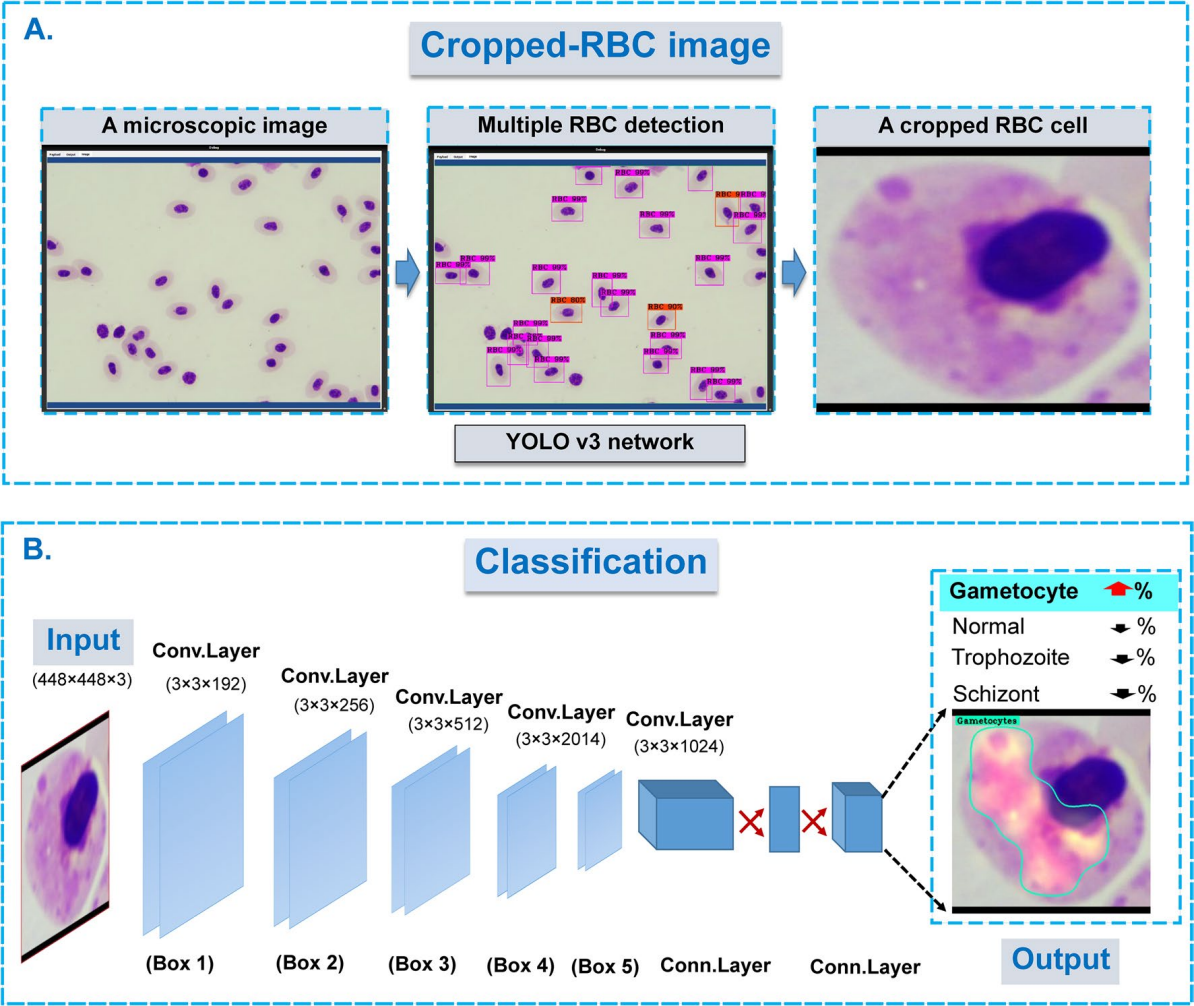
The schematic of the two-state learning strategies for classification of the single chicken’s RBC infected by *P. gallinaceum*. (**A**) A microscopic image with multiple RBC infected malaria stages was captured under oil immersion field (×1000 magnification). An individual RBCs were cropped under CiRA CORE platform if these cells were correctly detected by well-trained YOLO v3 model. (**B**) Each cropped-RBC cell with/without malaria infection was then classified by trained image classification networks including Darknet, Darknet19, Darknet19-448 and Densenet201, respectively. In the extracting hierarchical features, feature (attention) maps was generated by filters of each convolution layer (within the feature extractor part) and used for visualization through channel-wise averaged gradients. The attention map of the JET color gradient is used to visualize self-region of the interest of the input based on optimal weight. [the figure is drawn by Microsoft PowerPoint. https://www.microsoft.com/en-us/microsoft-365/powerpoint].

Two datasets were developed by a team of experts who labeled all 432 microscopic examination images for RBCs^1,39^. The first dataset is obtained from microscopic images of chicken RBCs scattered around the whole image. A rectangular bounding box closely fitting to the RBC image region was manually designated for each individual RBC. The ground truth data file was saved as a collection of RBCs with designated bounding boxes. The first dataset was then set as the ground truth file for the object detection model (YOLOv3). The ground truth was deposited as shown in this link as followed; https://git.cira-lab.com/cira-medical/classification-of-avian-malaria-parasite. Using the first dataset, the YOLOv3 model was subsequently employed to learn and detect individual RBC. By feeding all microscopic images to the trained YOLOv3, the model output reported all the image regions that contained any chicken's RBC. Where an RBC was detected, the image region of a single RBC (both normal and parasitized-RBCs) was cropped using the standard image crop technique. Each single RBC image was then used for the preparation of the second dataset (Fig. 1).

The deep learning classification model was used in the second stage to identify a specific relative class within the cropped image extracted from the captured image containing a single chicken's RBC. The second dataset consists of 12,761 cropped images containing a significant proportion of regions of interest (ROI). Pooled single cropped-cell images were grouped and assigned labels according to four classes based on their physiological and morphological stages, including: (i) normal RBC for 6724, (ii) trophozoite for 5343, (iii) schizont for 657, and (iv) gametocyte for 37, respectively. Each class above was randomly divided into training (90 per cent) and testing (10 per cent) sets, minimizing potential similarities between these two sets. This protocol can be trustable for preventing sample selection bias from the same patient’s slides. While disproportionate sample size between classes has been identified and can trigger biased-prognosis against a class with a large number of image sets, a deep learning approach with multi-layered layers and data annotation can be explored prior to model training. To speed up model convergence, rescale the image from raw pixels to 448 × 448 pixels before training with a chosen neural network model.

### Data augmentation and model training

Data augmentation techniques were introduced to the dataset prior to training to avoid over-fitting of the training image collection, and it was also used in the case of an unbalanced class. The technique involves rotations, brightness/contrast, blurriness and Gaussian noise under the following conditions:

(i) The rotational angle was performed with the degree value specified from − 180° to 180°, varied at every 45°.
(ii) The brightness and contrast were adjusted by multiplying all pixel values for seven steps, ranging from 0.4 to 1.6.
(iii) The Gaussian noise distribution was applied to help the model to distinguish an original image from the annotated image with noise. We used 5 steps accompanying to 5-standard deviation (σ = 0, 4, 8, 12 16 and 20).
(iv) Gaussian blur was used to vary the image’s sharpening to blurring with 5 steps (Gaussian filter) with a standard deviation of 5, ranging from 1 to 10.

These models mentioned above were trained on the in-house deep learning platform CiRA CORE (https://git.cira-lab.com/cira/cira-core), under Ubuntu version 16.04, 16 GB RAM, and NVIDIA GeForce GTX1070Ti graphic processor unit. Furthermore, the qualified models were trained for at least 100,000 epochs in order to record the learned parameters. The likelihood of a threshold greater than and equal to 50% is considered to be a true positive value, which incurs no cost^42,44^. Otherwise, the result of image classification would produce false positive values that are unexpected in medical diagnosis.

### Model evaluations

The performance quality of the model- and class-wise prediction was evaluated in terms of the following statistical metric parameters: accuracy, sensitivity, specificity, precision and misclassification rate^11,42,47^.

1 $$Sensitivity=\frac{TP}{TP+FN},$$

2 $$Specificity=\frac{TN}{TN+FP},$$

3 $$Accuracy=\frac{TP+TN}{TP+TN+FP+FN},$$

4 $$Missclassification  \, rate=\frac{FP+FN}{TP+TN+FP+FN},$$

5 $$Precision= \frac{TP}{TP+FP},$$

where TP is the number of true positive values, TN is the number of true negative values, FP is the number of false positive values, and FN is the number of false negative values.

The confusion matrix table was created to demonstrate the overall accuracy of both the model capability in the multi-class classification. An area under the Receiver Operating Characteristic (ROC) curve (AUC) at 95% confident of interval (CI) was designed to calculate the average accuracy of the model using python software.

## Results

Accompanying those protocols in the method part, we combined YOLOv3 and image classification (Darknet/Densenet201) models in order to localize, identify and classify a single RBC cell from any microscopic examination image with multiple-RBCs. Their relative RBC’s classes were then classified as both normal and that infection varied based on pathological features (Supplementary Fig. S1). Besides, the hybrid platform of YOLOv2 with ResNet-50 detector helps improve the average precision of the proposed detector up to 81% compared to a previous single model^41^.

### Performance comparison of classification models

In this analysis, the model-wise performance was assessed as to whether the classification model was the best-selected model based on an attention map and used to estimate *P. gallinaceum*-infected blood phases (Supplementary Fig. S1). These models included Darknet, Darknet19, Darknet19-448 and Densenet201 as described above. For classification models for avian malaria parasite phases, performance metrics such as average precision, uncertainty confusion matrix table, and ROC curve were estimated and compared. In addition, we randomly split the single-cell images for training and compared the image sets to determine if the different models generated the same classifier performance. All well-trained models provided us with high average accuracy values of more than 97%, which increased to 99.2% for the Darknet algorithm (Table 1). In addition, all other two statistics also support the Darknet network as a superior performance at 99.2 per cent for both sensitivity and specificity, respectively (Tables 2, 3). Interestingly, all models used showed less than 2% error rate, which was particularly impressive considering that the Darknet network gave less than 1% error rate (Table 4). On average, the precision of the Darknet also showed outstanding performance at 99.0% more than others (Table 5).

**Table 1:** Model-wise comparison and multiclass-wise comparison based on the accuracy from one-class versus total.

DCNNs	Average	Normal	Trophozoite	Schizont	Gametocyte
Darknet	0.992	0.983	0.983	1.000	1.000
Darknet19	0.991	0.981	0.981	1.000	1.000
Darknet19_448	0.984	0.967	0.967	1.000	1.000
Densenet201	0.979	0.963	0.957	0.994	1.000

**Table 2:** Model-wise comparison and multiclass-wise comparison based on the sensitivity from one-class versus total.

DCNNs	Average	Normal	Trophozoite	Schizont	Gametocyte
Darknet	0.992	0.974	0.992	1.000	1.000
Darknet19	0.991	0.972	0.990	1.000	1.000
Darknet19_448	0.982	0.972	0.957	1.000	1.000
Densenet201	0.977	0.966	0.940	1.000	1.000

**Table 3:** Model-wise comparison and multiclass-wise comparison based on specificity from one-class versus total.

DCNNs	Average	Normal	Trophozoite	Schizont	Gametocyte
Darknet	0.992	0.993	0.976	1.000	1.000
Darknet19	0.992	0.991	0.975	1.000	1.000
Darknet19_448	0.984	0.962	0.975	1.000	1.000
Densenet201	0.981	0.960	0.969	0.994	1.000

**Table 4:** Model-wise comparison and multiclass-wise comparison based on misclassification rate from one-class versus total.

DCNNs	Average	Normal	Trophozoite	Schizont	Gametocyte
Darknet	0.009	0.017	0.017	0.000	0.000
Darknet19	0.010	0.019	0.019	0.000	0.000
Darknet19_448	0.017	0.033	0.033	0.000	0.000
Densenet201	0.022	0.037	0.043	0.006	0.000

**Table 5:** Model-wise comparison and multiclass-wise comparison based on precision from one-class versus total.

DCNNs	Average	Normal	Trophozoite	Schizont	Gametocyte
Darknet	0.990	0.993	0.968	1.000	1.000
Darknet19	0.990	0.992	0.966	1.000	1.000
Darknet19_448	0.983	0.966	0.965	1.000	1.000
Densenet201	0.980	0.964	0.956	0.896	1.000

**Figure 2:**
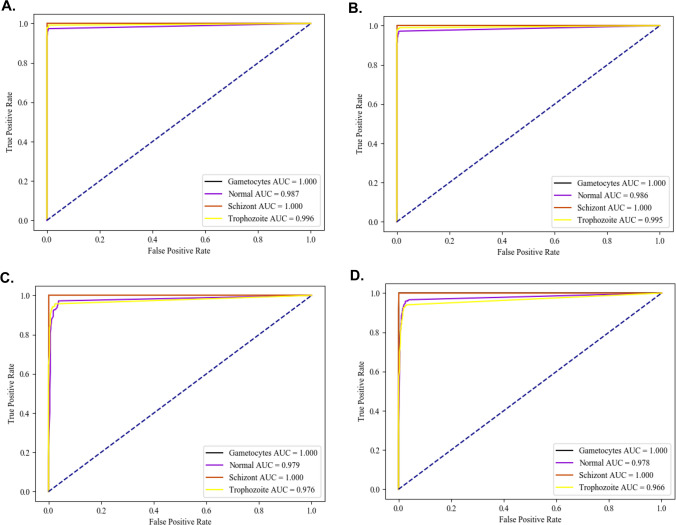
The AUC under the ROC curve analyzed by the model- wise performance following; (**A**) Darknet, (**B**) Darknet19, (**C**) Darknet19_448 and (**D**) Densenet201, respectively. Besides, the plot also present class-wise comparison following; Gametocyte, Normal RBC, Schizont and Trophozoite, respectively. All images were plotted under the CiRA CORE plug-in encoded by using Python 3.5.0 (https://launchpad.net/~deadsnakes/+archive/ubuntu/ppa), matplotlib 3.4.2 (https://pypi.org/project/matplotlib/) and pandas 1.3.1 (https://pypi.org/project/pandas/).

General accuracy, obtained from the confusion matrix table, showed outstanding values for all four models (Table 6). In addition, the Darknet model outperformed all other models by more than 97% (Table 6(1)–(4)). Overall, the general accuracy from the confusion matrix gave greater than 95 per cent, except for the Darknet19-448 for classifying the normal RBC class and followed by the DenseNet201 for classifying the trophozoite class gave us at 91.21 and 94.01 per cent, respectively (Table 6(3), (4)). Specifically, this manifested in an overall AUC ranking of 0.986–1.000 (Fig. 2). Hence, the Darknet models can reproduce superior performance in the classification of the development phases of *P. gallinaceum* in every other model architecture.

**Table 6:** Comparison of the studied model performance using the confusion matrix table.

Darknet	Normal	Trophozoite	Schizont	Gametocyte
**(1) Darknet model**				
Normal	594 (97.38%)	4 (0.83%)	0 (0.00%)	0 (0.00%)
Trophozoite	16 (2.62%)	480 (99.17%)	0 (0.00%)	0 (0.00%)
Schizont	0 (0.00%)	0 (0.00%)	60 (100.00%)	0 (0.00%)
Gametocyte	0 (0.00%)	0 (0.00%)	0 (0.00%)	5 (100.00%)

Darknet19	Normal	Trophozoite	Schizont	Gametocyte
**(2) Darknet19 model**				
Normal	593 (97.21%)	5 (1.03%)	0 (0.00%)	0 (0.00%)
Trophozoite	17 (2.79%)	479 (98.97%)	0 (0.00%)	0 (0.00%)
Schizont	0 (0.00%)	0 (0.00%)	60 (100.00%)	0 (0.00%)
Gametocyte	0 (0.00%)	0 (0.00%)	0 (0.00%)	5 (100.00%)

Darknet19-448	Normal	Trophozoite	Schizont	Gametocyte
**(3) Darknet19-448 model**				
Normal	593 (91.21%)	21 (4.34%)	0 (0.00%)	0 (0.00%)
Trophozoite	17 (2.79%)	463 (95.66%)	0 (0.00%)	0 (0.00%)
Schizont	0 (0.00%)	0 (0.00%)	60 (100.00%)	0 (0.00%)
Gametocyte	0 (0.00%)	0 (0.00%)	0 (0.00%)	5 (100.00%)

Densenet201	Normal	Trophozoite	Schizont	Gametocyte
**(4) Densenet201 model**				
Normal	589 (96.56%)	22 (4.55%)	0 (0.00%)	0 (0.00%)
Trophozoite	21 (3.44%)	455 (94.01%)	0 (0.00%)	0 (0.00%)
Schizont	0 (0.00%)	7 (1.45%)	60 (100.00%)	0 (0.00%)
Gametocyte	0 (0.00%)	0 (0.00%)	0 (0.00%)	5 (100.00%)

### Multiple class comparison

Based on the best-selected network model, an interpretation of the classification outcome produced an empirical result in distinguishing the malaria parasite stages. Despite training the model with disproportionate sample sizes across classes, the neural network model's multiple class-wise consistency demonstrated great statistical values for all classes, with greater than 99% of all output matrices used showing no bias against either class (Tables 1, 2, 3). This is because our best-chosen model can distinguish malaria phases with high precision but a low rate of misclassification of less than 1% found (Tables 4, 5). This may be one of the benefits of applying the class-balancing data augmentation protocol to the prepared dataset. This study's findings indicate that the model could be validated using multiple blood smears derived from real-world contexts.

## Discussion

In this study, the robustness of deep neural network models led to the discovery of a new approach for more rapid screening of avian malaria under a microscope. Asymptomatic diseases, in particular, can lead to disease transmission and even death if not adequately prevented^48,49^. This study may contribute to the main comparison of several image classification models based on images of avian malaria, *P. gallinaceum*. Also, both qualitative and quantitative data were used to evaluate the performance of the proposed models.

Several CNNs have also been developed to provide a number of pre-trained ImageNet classification models, including AlexNet, Darknet, VGG-16, Resnet^50^, ResNext, and Densenet201^51^, for use as effective in image classification applications. According to an evaluation of the performance of these pre-trained models, the Darknet model has a higher accuracy than the Densenet201 model and also provides the fastest processing time in both CPU and GPU^45,52^. Even though we trained network models with actual patient-level yet unbalanced class sizes, the performance of well-trained proposed models has shown an outstanding outcome based on several statistical parameters, even in a multi-class comparison. Since the project was a significant success, this would help to advance the development of innovative technologies in order to implement and validate them in a real-world environment.

We would like to illustrate our approach to developing deep learning models from concatenated neural network architectures to make clinical applications by viewing the video as follows: https://youtu.be/--pzvwSrUdc. Nonetheless, before the technology is implemented, the inter- and intra-variability examinations should be performed in order to validate the degree of consensus between the predictive model's functional performance metrics and the human expert^53^.

The limitation of the study is deeply based on the determination of three key points for the preparation of the dataset^54^, including; (i) differences in the parasite-blood stages^24^, (ii) infection status that can induce either single or multiple infections, and (iii) co-infections in any single blood cell. It is worth noting that in this study, only fully grown gametocyte were counted as gametocytes because young gametocytes are transformed from late trophozoites. The imbalance data is another consequence of dataset preparation, though it is less severe than the others listed above. In the case of differences in the blood stage of each parasite, an increasing number of samples is a potential solution. The analysis has influenced model efficiency under the research design. This may be because the image set is a special attribute between classes that helps to increase class differentiation for the well-trained models. We have different sizes of test set including 610, 484, 60 and 5 images for normal RBC-, trophozoite-, schizont-, and gametocyte classes, respectively. Although our datasets used are imbalanced, in the study, the overall accuracy shows more than 98–100% in classifying between the independently different classes. Since our models were trained utilizing the well-planned dataset preparation described in the method section, the role of data augmentation and data balancing for environmental simulation improved the classification efficiency^47^. Additionally, five-fold cross validation was also studied to help confirm whether our selected model gave consistently general accuracy and no predictive bias^54,55^. According to our results, cross-validation solves the problem of overfitting. This is because the cross-validation applied can help minimize the cross-validated error to build the optimal model and result in indifferent statistical parameters used between any experiment (Supplementary Tables S1, S2). Furthermore, we appropriately chose an image of a single species of parasite infection to be examined. Since the staining of artifacts/impurities of uninfected blood cells can interfere with the prediction, the expert’s simple staining of color under the Giemsa staining process also provides us with the ideal dataset^56^. It is important to note that all images to be included in this application should be prepared properly, otherwise the abnormal host cells such as segmented erythrocyte nuclei or other debris might lead to false identification. Confirmation by a technician is still necessary. Nevertheless, the model must be validated in a specific environment of multiple pathogens and co-infections, which could already exist in field samples.

## Conclusions

In this study, the application of a deep neural learning network to identify and verify avian malaria and to assess it by using truly invisible images that have never been published. Based on our results, we demonstrated the superior performance of a novel hybrid two-stage model (object detection YOLOv3 and Darknet/Densenet201 classification algorithms). Although we trained network models with real patient-level yet unbalanced class sizes, the performance of well-trained proposed models was exceptional based on many statistical parameters, including in a multi-class comparison. Furthermore, we can deduce that only a model learning methodology and data preparation should be used in the research design. Fortunately, the study showed a high value of the various statistical measurements listed above, which could be useful for cytological technicians in making decisions. It would be useful to diagnose malaria infections wherever there is due to a shortage of molecular biology and veterinary knowledge.

## Supplementary Information

Supplementary Video 1.

Supplementary Tables.

Supplementary Figure 1.

## Data Availability

The data that support the findings of this study are available from the corresponding author’s GitHub repository: URL: https://git.cira-lab.com/cira-medical/classification-of-avian-malaria-parasite.
