# Supplementary material for: Classification for avian malaria parasite Plasmodium gallinaceum blood stages by using deep convolutional neural networks
Source: Sci Rep. 2021 Aug 19;11:16919. doi: 10.1038/s41598-021-96475-5 (PMC8376898; doi:10.1038/s41598-021-96475-5)
Supplement: Supplementary file 2 — Supplementary Tables. [file 41598_2021_96475_MOESM2_ESM.docx]

**Classification for avian malaria parasite *Plasmodium gallinaceum* blood stages by using deep convolutional neural networks**

**Authors**

Veerayuth Kittichai^1^, Morakot Kaewthamasorn^2^, Suchansa Thanee^2^, Rangsan Jomtarak^3^, Kamonpob Klanboot^4^, Kaung Myat Naing^4^, Teerawat Tongloy^4^,

Santhad Chuwongin^4^, Siridech Boonsang^5*^

**Affiliations**

^1^Faculty of Medicine, King Mongkut Institute of Technology Ladkrabang, Bangkok, Thailand.

^2^Veterinary Parasitology Research Unit, Faculty of Veterinary Science, Chulalongkorn University, Bangkok, Thailand.

^3^Faculty of Science and Technology, Suan Dusit University, Bangkok, Thailand.

^4^College of Advanced Manufacturing Innovation, King Mongkut Institute of Technology Ladkrabang, Bangkok, Thailand.

^5^Department of Electrical Engineering, Faculty of Engineering, King Mongkut Institute of Technology Ladkrabang, Bangkok, Thailand

*Correspondence author

Email: [Siridech.bo@kmitl.ac.th](mailto:Siridech.bo@kmitl.ac.th)

**Table S1. Dataset used for studying the 5-fold cross validations.** Sample size of scizont and gametocyte classes (data shown in parentheses) were increased under data augmentation function that used to reduce dataset bias.

| **Folds** | **Normal** | **Trophozoite** | **Schizont** | **Gametocyte** |
| --- | --- | --- | --- | --- |
| 1 | 4,891 | 3,887 | 657 (7,642) | 32 (3,789) |
| 2 | 4,891 | 3,887 | 657 (7,642) | 32 (3,789) |
| 3 | 4,891 | 3,887 | 657 (7,642) | 32 (3,789) |
| 4 | 4,891 | 3,887 | 657 (7,642) | 32 (3,789) |
| 5 | 4,891 | 3,887 | 657 (9,552) | 32 (3,789) |
| Total | 24,455 | 19,435 | 3285 (40,120) | 160 (18,945) |

**Table S2. Performance of 5-fold cross validation**. The model performance were evaluated using statistical parameters including: accuracy, sensitivity, specificity, precision, RUC’s AUC and misclassification rate.

| **k-folds** | **Data** | **Accuracy** | **Misclassification Rate** | **Sensitivity** | **Specificity** | **Precision** | **AUC** |
| --- | --- | --- | --- | --- | --- | --- | --- |
| Fold-1 | Normal | 0.953 | 0.047 | 0.854 | 0.984 | 0.946 | 0.921 |
|  | Trophozoite | 0.95 | 0.05 | 0.916 | 0.958 | 0.839 | 0.953 |
|  | Schizont | 0.994 | 0.006 | 0.999 | 0.991 | 0.985 | 0.999 |
|  | Gametocytes | 0.999 | 0.001 | 1.000 | 0.999 | 0.997 | 0.895 |
|  | **Average** | **0.974** | **0.026** | **0.942** | **0.983** | **0.942** | **0.942** |
| Fold-2 | Normal | 0.982 | 0.018 | 0.976 | 0.984 | 0.952 | 0.987 |
|  | Trophozoite | 0.975 | 0.025 | 0.928 | 0.986 | 0.940 | 0.963 |
|  | Schizont | 0.992 | 0.008 | 0.984 | 0.997 | 0.995 | 0.992 |
|  | Gametocytes | 1.000 | 0.000 | 1.000 | 1.000 | 0.998 | 0.888 |
|  | **Average** | **0.987** | **0.013** | **0.972** | **0.992** | **0.971** | **0.958** |
| Fold-3 | Normal | 0.963 | 0.037 | 0.966 | 0.962 | 0.891 | 0.979 |
|  | Trophozoite | 0.936 | 0.064 | 0.857 | 0.955 | 0.819 | 0.921 |
|  | Schizont | 0.969 | 0.031 | 0.932 | 0.991 | 0.985 | 0.965 |
|  | Gametocytes | 0.990 | 0.010 | 0.950 | 1.000 | 0.998 | 0.878 |
|  | **Average** | **0.965** | **0.035** | **0.926** | **0.977** | **0.923** | **0.936** |
| Fold-4 | Normal | 0.969 | 0.031 | 0.933 | 0.981 | 0.939 | 0.963 |
|  | Trophozoite | 0.957 | 0.043 | 0.908 | 0.969 | 0.872 | 0.948 |
|  | Schizont | 0.987 | 0.013 | 0.973 | 0.996 | 0.993 | 0.986 |
|  | Gametocytes | 0.998 | 0.002 | 0.998 | 0.998 | 0.992 | 0.838 |
|  | **Average** | **0.978** | **0.022** | **0.953** | **0.986** | **0.949** | **0.934** |
| Fold-5 | Normal | 0.969 | 0.031 | 0.933 | 0.981 | 0.939 | 0.963 |
|  | Trophozoite | 0.957 | 0.043 | 0.908 | 0.969 | 0.872 | 0.948 |
|  | Schizont | 0.987 | 0.013 | 0.973 | 0.996 | 0.993 | 0.986 |
|  | Gametocytes | 0.998 | 0.002 | 0.998 | 0.998 | 0.992 | 0.838 |
|  | **Average** | **0.978** | **0.022** | **0.953** | **0.986** | **0.949** | **0.934** |

**Figure S1**. **Prediction labeling of the outcome through all four selectable models**. The color of the attention map indicated the field of interest predicted by each model. The X-axis is the input image and following the study models such as Darknet, Darknet19, Darknet19-448 and Densenet201. The Y-axes is representative for individual chicken-RBCs that are regular and those that are infected malaria, including trophozoites, schizonts and gametocytes**.**

**Supplementary video file.** The proposed technique for classifying the blood stages of avian malaria, *P. gallinaceum*, using hybrid two stage models (object identification YOLOv3 and Darknet classification algorithms). The first stage of the proposed model is the YOLOv3-based object detection, which aims to distinguish a single RBC image from those inside a microscopic image. Among other object detection models, the YOLOv3 model outperformed the others, in terms of localization and classification accuracy. Cropped images (single RBC) from its first stage model inference were used as inputs for the second stage model. The classification model was used in the second stage to categorize the single RBC detected. Darknet model, a top-5 pre-trained model reproducing accuracy at 94.7%, was used for studying the image-classification. The model prediction was automatically masked and colored with a JET color map generated by the Class Activation Map (CAM) algorithm.
