# Supplementary figures and images for: Classification for avian malaria parasite Plasmodium gallinaceum blood stages by using deep convolutional neural networks
Source: Sci Rep. 2021 Aug 19;11:16919. doi: 10.1038/s41598-021-96475-5 (PMC8376898; doi:10.1038/s41598-021-96475-5)

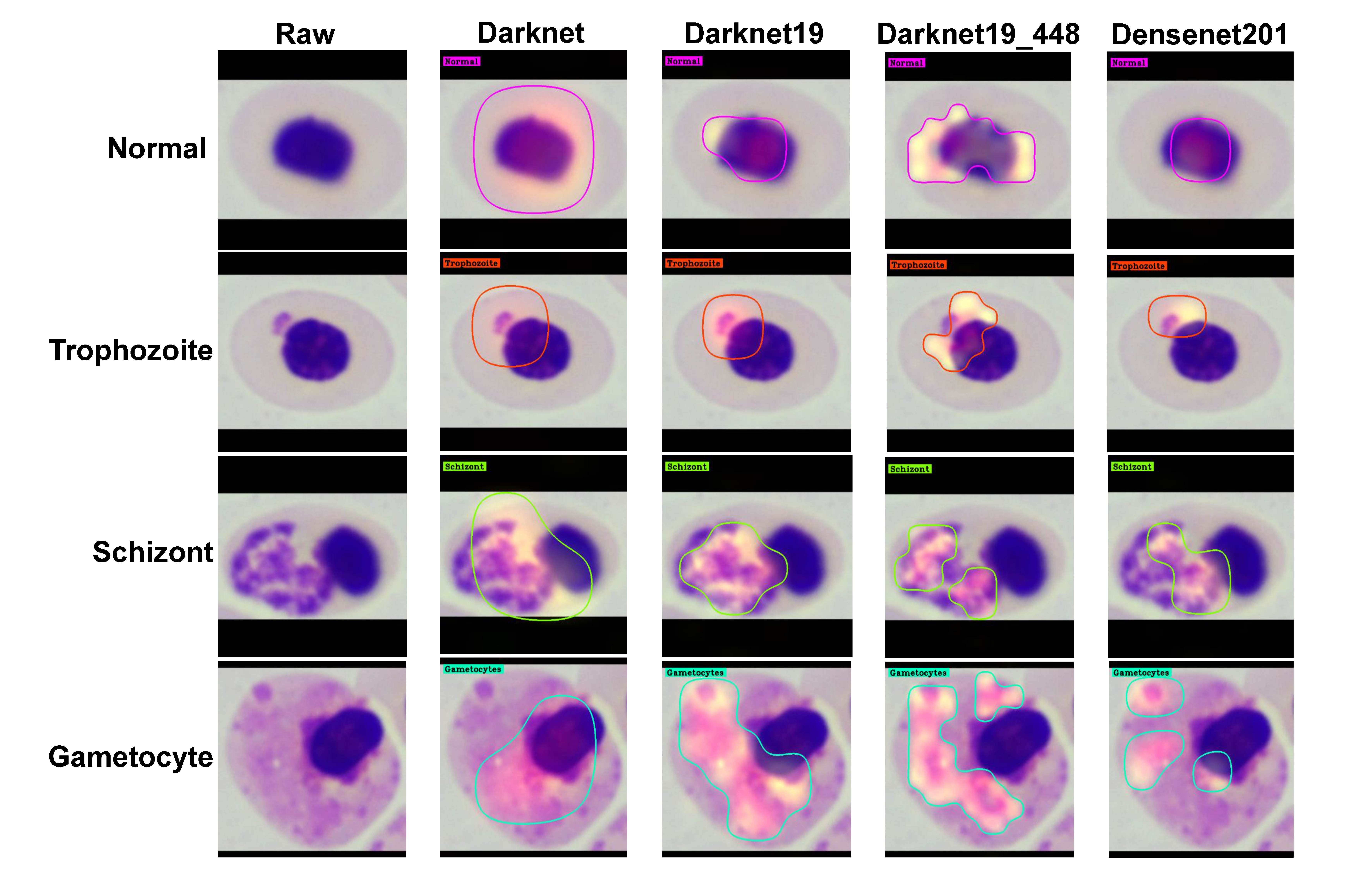

Supplement: Supplementary file 3 — Supplementary Figure 1. [file 41598_2021_96475_MOESM3_ESM.tiff]
